# Restricted Social Engagement among Adults Living with Chronic Conditions

**DOI:** 10.3390/ijerph15010158

**Published:** 2018-01-19

**Authors:** Kayla P. Meek, Caroline D. Bergeron, Samuel D. Towne, SangNam Ahn, Marcia G. Ory, Matthew Lee Smith

**Affiliations:** 1 College of Public Health, The University of Georgia, Athens, GA 30602, USA; kpmeek@uga.edu; 2 Bexar County Community Health Collaborative, San Antonio, TX 78212, USA; caroline.bergeron@healthcollaborative.net; 3 Center for Population Health and Aging, Texas A&M University, College Station, TX 77843, USA; towne@sph.tamhsc.edu (S.D.T.J.); sahn@memphis.edu (S.A.); mory@sph.tamhsc.edu (M.G.O.); 4 School of Public Health, Texas A&M University, College Station, TX 77843, USA; 5 Southwest Rural Health Research Center, Texas A&M University, College Station, TX 77843, USA; 6 School of Public Health, The University of Memphis, Memphis, TN 38152, USA

**Keywords:** aging, chronic disease, disease management, social isolation, socialization, intervention

## Abstract

*Background*: Social engagement is key to health and quality of life. Little is known about social engagement patterns of middle-aged and older adults who live with one or more chronic illnesses. This study investigated social engagement restrictions among middle-aged and older adults with chronic conditions and factors associated with these restrictions. *Methods*: Cross-sectional representative data from the National Council on Aging Chronic Care Survey were examined for relationships between social engagement restrictions and chronic conditions, health status, support, quality of life implications, self-care barriers, caregiving, and demographics. Associations were tested using bivariate analyses and binary logistic regression. *Results*: Participants were 793 middle-aged (age 44–64) and older adults (age 65+) with one or more chronic conditions. Factors associated with social engagement restrictions included having higher education, receiving care, having more physician visits and hospitalizations, being disabled, being unemployed, and having higher Emotional and Physical Problems Scale scores. *Conclusions*: Findings reveal the prevalence of social engagement restrictions among middle-aged and older adults with chronic conditions. Results highlight the importance of promoting research, assessments, and interventions to increase social engagement among this aging population.

## 1. Introduction

Chronic diseases such as cancer and diabetes are currently the leading causes of death and disability in the United States (U.S.) [1] and worldwide [2]. More than 90% of older adults report having at least one chronic condition, with over 70% reporting at least two chronic conditions [3]. While having a chronic disease can affect an individual’s ability to engage in social interactions [4], social engagement among older adults can prevent their condition from progressing into a disability [5]. Social engagement, including social participation in specific activities, social network, and social support [6], may promote resources that enhance self-efficacy in disease management and resilience to disability [7]. Gallant [8] reported that social influences can have both positive and negative effects on chronic disease self-management. Social ties can provide instrumental and emotional support, which are positively associated with better self-management behaviors [8] as well as increased mental and physical health and quality of life [9]. Conversely, those with a chronic disease may need to manage social influences (e.g., calming family members, dealing with unhelpful advice) [8], which may hinder self-management and cause intentional limited contact with others [8].

A discussion of chronic disease merits mention of potential solutions as well. Chronic diseases are multifaceted issues, which require multi-level approaches to help individuals manage their conditions and prevent negative consequences (e.g., rapid disease progression, hospitalization). Interventions of potential use may include both behavioral (e.g., Chronic Disease Self-Management Program) [10] and environmental or built environment interventions (e.g., walkable communities) [11,12]. However, an important step in targeting interventions is to first identify factors associated with how social engagement may affect adults living with chronic disease. This line of research will allow for tailored interventions to those most at-risk.

**Figure 1:**
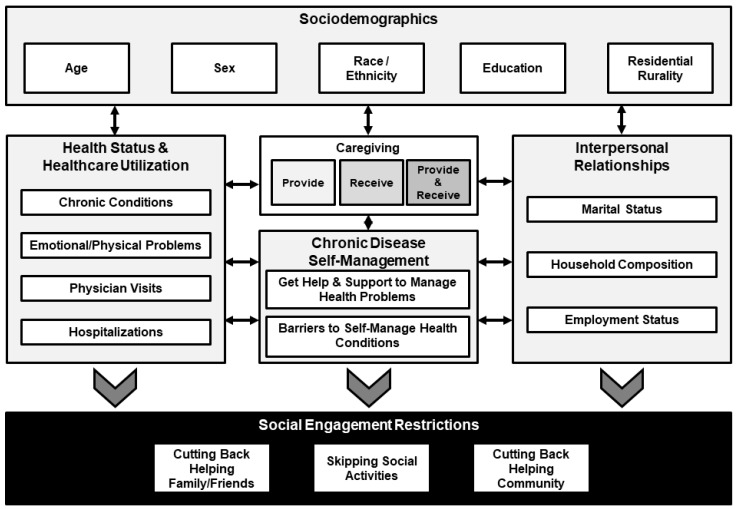
Conceptual framework.

Researchers have examined the health benefits of social engagement by gender and age [13], race/ethnicity [5], marital status [14], caregiver status [15], disability [5], and hospitalization rates [16]. However, limited research examines the impact of these and other factors (e.g., education, rurality, household composition, and employment status) on social engagement among individuals living with chronic diseases. Restricting one’s social engagement, for intentional or unintentional reasons, may lead to social isolation [17], limited physical and cognitive ability over time [18], and an increased risk of death [19]. The purposes of this study were to identify the prevalence of social engagement restrictions among middle-aged and older adults with chronic conditions and examine factors associated with reporting social engagement restrictions. This study is unique in that few studies have examined health-related restricting social behaviors of middle-aged and older adults with one or more chronic conditions. This is especially true among a diverse, national sample self-reporting an array of chronic condition types. Further, this study is unique in that it includes middle-aged and older adults, which offers inclusivity of those with different disease progressions and social structures and responsibilities. Findings from this study have implications to identify risk and reduce engagement restrictions through intervention engagement to promote healthy aging. Figure 1 illustrates the conceptual framework used to guide this study.

## 2. Materials and Methods

### 2.1. Study Data and Measures

Study data were from the National Council on Aging (NCOA) Chronic Care Survey, a nationally-representative probability survey of Americans 44 years and older with chronic conditions conducted by Lake Research Partners [20]. The survey employed telephone-based interviewing to collect data using random digit dialing sampling techniques. Telephone interviews were conducted in English and Spanish. The response rate was estimated to be 86.6%. Data were weighted by age, race, and region to reflect the overall American population aged 44 years and older with one or more chronic conditions. The sampling error margin was estimated to be ±2.9% [20]. Additional details regarding the survey methodology is available in other published literature [21,22,23,24,25].

Eligible participants reported having at least one chronic condition at the time of the study. Participants were screened for chronic condition(s) with the following question(s): “Have you ever been told by a doctor, nurse or other health professional that you have [name of chronic condition]?” Chronic conditions included heart disease, cancer, stroke, diabetes, arthritis, asthma, hypertension or high blood pressure, emphysema, chronic bronchitis, depression, anxiety, and others. Only participants who reported “yes” to at least one of these items were included in the survey [20]. Data were analyzed from 793 community-dwelling men and women 44 years and older with at least one chronic condition across the U.S. [20].

**Dependent variables**. Participants were asked to self-report some of the social engagement restrictions caused by coping with their health problem(s). Participants were asked: “As a result of your health problem(s), in the last 12 months ___?” Questions included: “have you had to cut down or skip any social activities?”, “have you had to cut back on helping family and friends?”, and “have you had to cut back on helping in your community, church, or volunteering in other ways?” The results were scored as “yes” or “no”. Each of these dichotomous items was examined independently. Then, the three dichotomous items were summed to determine the number of social engagement restrictions reported by each participant. This count variable was then dichotomized to indicate whether or not participants reported no social engagement restrictions or one or more social engagement restrictions.

**Self-reported chronic conditions**. From a list of conditions, participants were asked to self-report the types of chronic diseases that they had been diagnosed with. For ease of analysis, categories were collapsed into six chronic condition types including: cardiovascular disease (i.e., hypertension, heart disease, and stroke); diabetes; arthritis; lung disease (i.e., asthma, chronic bronchitis, and emphysema); depression or anxiety; and cancer. The number of endorsed disease types was also summed to create a continuous variable of the number of chronic conditions. Responses ranged from 1 to 6.

**Health status indicators**. Participants were asked to self-report aspects of their current health status using the number of physician visits and hospitalizations in the previous 12 months. Participants were asked, “In the past 12 months, how many times have you, yourself made a doctor visit?” Additionally, participants were asked, “In the past 12 months, how many times have you, yourself had an overnight stay in a hospital?” Possible responses ranged from 0 to 10 times for both of these open-ended items.

**General support perceptions**. Participants were asked to self-report their perceptions about receiving general support to manage their health problems. Participants were asked: “How often do you feel you get the help and support you need to improve your health and manage your health problems?” Responses were scored using a 5-point Likert-type scale ranging from “never” (scored 0) to “always” (scored 4).

**Quality of life implications**. Participants were asked to self-report some of the emotional and physical problems of coping with their health problems. Participants were asked: “As a result of your health problems, how often would you say you feel___?” Categories of interest included depressed or unhappy, angry, a lack of control, stressed, tired or lacking energy, and in physical pain. Responses were scored on a 4-point Likert-type scale ranging from “always” to never”. All items were summed to create a single composite score, the Emotional and Physical Problems Scale (EPPS) (Cronbach’s alpha = 0.629). Scores could range from 0 to 24, with higher scores indicating worse effects of dealing with emotional and physical problems.

**Self-care barriers**. Participants were asked to self-report their perceived barriers to self-care. Participants were asked to rate their level of agreement with the following statement: “I need help learning how to take better care of my health in a way that works for me and my life”. Responses were scored on a 4-point Likert-type scale ranging from “strongly disagree” to “strongly agree”. Based on the frequency distribution, participant responses were then dichotomized into two categories: “disagree” and “agree”.

**Caregiving**. Participants were asked to self-report their caregiver status. Participants were asked: “There are situations where people provide regular care or assistance to a family member or friend who is elderly, has a long-term illness, or disability. During the past month, did you provide any such care or assistance to a family member or friend?” Additionally, participants were asked: “During the past month, did you receive any such care or assistance from a family member or friend as a result of your health problems?” Response categories for these two items were “yes” and “no”. These two variables were combined to create a 4-category variable for care status. Response categories for this dependent variable included “neither give nor receive care”, “give care only”, “receive care only”, and “both give and receive care”.

**Sociodemographics**. Sociodemographic variables in this study included: age group (i.e., 44–64 years, 65+ years); sex (i.e., male, female); race/ethnicity (i.e., non-Hispanic white, non-Hispanic African American, Hispanic, other); education level (i.e., less than high school, high school, some college, college graduate, graduate school); marital status (i.e., unmarried, married); work status (i.e., employed, unemployed, retired, disabled); and the number of adults in the household.

### 2.2. Statistical Methods

All statistical analyses for this study were performed using SPSS (version 24, IBM Corporation, Armonk, NY, USA). Frequencies were calculated to examine the distribution of independent variables based on whether or not they reported one or more social engagement restriction. Significant differences for categorical variables were identified using Pearson’s chi-square tests. Significant differences for continuous and count variables were identified using independent sample *t*-tests. A series of four binary logistic regression models were fitted to identify the relative contribution of independent variables on social engagement restrictions. One model was fitted for each of the three social engagement restrictions independently (i.e., not having the restriction served as the referent group). Then, a model was fitted to examine factors associated with reporting one or more social engagement restriction (i.e., reporting no restrictions served as the referent group). An alpha < 0.05 was used to determine statistical significance for all analyses.

## 3. Results

Sample characteristics of the study participants are presented in Table 1. Of the 793 participants, 66% were between the ages of 44 and 64 years and 34% were aged 65 years and older. Respondents were disproportionately non-Hispanic white (84.0%) and married (62.2%). Participants reported having an average of 2.04 (±1.06) chronic conditions, 3.14 (±1.95) physician visits in the past 12 months, and 0.34 (±0.99) overnight hospitalizations in the past 12 months. Over 65% of participants reported that they did not need help learning how to take care of themselves. On average, participants reported less than one social engagement restriction (0.85 (±1.81)). Over 60% of participants reported no restrictions; 11.5% reported one restriction, 10.5% reported two restrictions, and 17.6% reported three restrictions. The most frequent restriction reported was skipping social activities (31.0%) followed by helping community/volunteering (27.3%) and helping family and friends (26.9%).

Personal characteristics by social engagement restrictions are presented in Table 1. A larger portion of participants who reported social engagement restrictions from their disease also reported that they received care, were disabled, and were unmarried. A larger proportion of participants who reported social engagement restrictions from their disease reported having diabetes, arthritis, lung disease, and depression. On average, participants who reported social engagement restrictions from their disease reported more chronic conditions, physician visits, and overnight hospitalizations. On average, those who reported social engagement restrictions from their disease had a higher EPPS score. A larger proportion of participants who reported social engagement restrictions from their disease reported needing assistance learning how to take care of themselves.

**One or more social engagement restrictions**. Table 2 displays the results of the logistic regression analysis explaining the social engagement restriction results from disease. Relative to the participants who neither provided nor received care, those who only received care were significantly more likely to report one or more social engagement restrictions (OR = 6.10, *p* < 0.001). Relative to the participants who had less than a high school education, those who had graduated high school (OR = 3.57, *p* = 0.005), had some college (OR = 4.25, *p* = 0.002), or were college graduates (OR = 4.61, *p* = 0.001) were significantly more likely to report one or more social engagement restrictions. For each additional physician visit (OR = 1.26, *p* < 0.001) reported by a participant, the individuals’ odds of reporting one or more social engagement restrictions increased. Relative to the participants who were employed, those who were unemployed (OR = 2.42, *p* = 0.005) and disabled (OR = 9.58, *p* < 0.001) were significantly more likely to report one or more social engagement restrictions. For each additional increase in the EPPS, a participant’s odds of reporting one or more social engagement restrictions increased (OR = 1.42, *p* < 0.001).

**Cut back family/friends**. Table 2 displays the results of the logistic regression analysis explaining a participant’s cut back on helping family and friends. Relative to the participants who neither provided nor received care, those who only received care were significantly more likely to report one or more social engagement restrictions (OR = 5.18, *p* < 0.001). Relative to the participants who had less than a high school education, those who had some college were significantly more likely to report cutting back on helping family and friends (OR = 2.57, *p* = 0.048). Relative to the participants who were employed, those who were unemployed (OR = 2.79, *p* = 0.002), retired (OR = 2.19, *p* = 0.013), or disabled (OR = 10.06, *p* < 0.001) were significantly more likely to report cutting back on helping family and friends. Compared to the participants who reported that they did not require help learning how to take care of themselves, those that reported that they need help learning to take care of themselves were significantly more likely to cut back on helping family and friends (OR = 1.67, *p* < 0.001). For each additional increase in the EPPS, a participant’s odds of reporting cutting back on helping family and friends increased significantly (OR = 1.36, *p* < 0.001).

**Cut back or skip social activities**. Table 2 displays the results of the logistic regression analysis explaining a participant’s cut back or skip of social activities. Relative to the participants who neither provided nor received care, those who only provided care (OR = 1.71, *p* = 0.025) and those who only received care (OR = 3.72, *p* = 0.001) were significantly more likely to report cutting down or skipping social activities. Relative to the participants who had less than a high school education, those who had education levels of high school or less (OR = 4.02, *p* = 0.003), some college (OR = 3.85, *p* = 0.006), were college graduates (OR = 5.94, *p* < 0.001), or attended graduate school (OR = 3.72, *p* = 0.019) were significantly more likely to report cutting down or skipping social activities. For each additional physician visit (OR = 1.34, *p* < 0.001) and hospitalization (OR = 1.40, *p* = 0.005) reported by a participant, their odds of reporting cutting down or skipping social activities increased. Relative to the participants who were employed, those who were disabled (OR = 6.25, *p* < 0.001) were significantly more likely to report cutting down or skipping social activities. Relative to the participants who reported never receiving the help and support needed to manage their health problems, those that reported that they rarely (OR = 3.5, *p* = 0.025), occasionally (OR = 3.39, *p* = 0.014), or frequently (OR = 2.77, *p* = 0.035) received the help and support needed to manage their health problems were significantly more likely to report cutting down or skipping social activities. For each additional increase in the EPPS, a participant’s odds of reporting cutting down or skipping social activities increased (OR = 1.29, *p* < 0.001).

**Cut back on helping in the community**. Table 2 displays the results of the logistic regression analysis explaining a participant’s cut back on helping in one’s community. Relative to the participants who neither provided nor received care, those who only received care were significantly more likely to report cutting back on helping in their community (OR = 3.42, *p* = 0.001). Relative to the participants who were between the ages of 44–64 years old, participants who were 65 years and older (OR = 1.70, *p* = 0.046) were significantly more likely to report cutting back on helping in their community. Relative to male participants, female participants (OR = 1.85, *p* = 0.004) were significantly more likely to report cutting back on helping in their community. Relative to the participants who had less than a high school education, those who had education levels of high school or less (OR = 3.63, *p* = 0.003), some college (OR = 2.83, *p* = 0.022), or were college graduates (OR = 3.76, *p* = 0.004), were significantly more likely to report cutting back on helping in their community. For each additional physician visit (OR = 1.16, *p* = 0.010) and hospitalization (OR = 1.26, *p* = 0.037) reported by a participant, their odds of reporting cutting back on helping in their community increased significantly. Relative to the participants who were employed, those who were unemployed (OR = 2.17, *p* = 0.015) and disabled (OR = 3.23, *p* = 0.001) were significantly more likely to report cutting back on helping in their community. For each additional increase in the EPPS, a participant’s odds of reporting cutting back on helping in their community increased significantly (OR = 1.30, *p* < 0.001).

## 4. Discussion

The current study examined social engagement restrictions among community-dwelling middle-aged and older adults with chronic diseases and investigated factors associated with three specific restrictions (i.e., skipping social activities, helping family and friends, and helping community/volunteering). While over 60% of participants reported having no social engagement restrictions, almost 18% of participants reported having all three restrictions. Findings suggest that a large proportion of middle-aged and older participants manage their chronic diseases and engagement in social relations [8]. Overall, factors associated with reporting one or more social engagement restrictions included having higher education, receiving care, having more physician visits and hospitalizations, being disabled, being unemployed, and having higher Emotional and Physical Problems Scale (EPPS) scores. Nuances in associated factors were identified based on the restriction type. As previously found, individuals living with multiple morbidity [26] and who are disabled [27] reported more social engagement restrictions. As suggested by this sum of physical impairments [28], individuals with both disease and disability are more likely to participate in less social activities than those managing a disease but not a disability [27]. Moreover, disabled individuals in general have lower social engagement [29] due to several barriers including emotional and psychological barriers, structural barriers, and potential discriminatory perceptions and attitudes by individuals who are not disabled [30].

One surprising finding was that middle-aged and older adults with more education were more likely to report one or more social engagement restrictions than their less educated counterparts. Educated participants restricted their own social engagement by cutting back on helping family and friends, attending social activities, skipping social activities, and helping the community. Education is generally recognized as a protective factor for isolation among middle-aged and older adults [31]; however, this case may reveal the important psychosocial nature of chronic disease self-management [32]. Highly educated participants may be restricting their activities to take care of themselves [20]; however, their social engagement restrictions may also be associated with an avoidance to be a burden on others [33], with a loss of social roles, independence, and potential self-worth [32], which may result in a loss of confidence in managing one’s own chronic condition [32], and an eventual downward health trajectory. Future qualitative research is needed to explore the conscious decisions of middle-aged and older adults with chronic conditions of various educational levels who choose to restrict or not restrict their social engagement.

Another surprising finding was that age was not directly associated with social engagement restrictions. While older age was associated with cutting back on helping in the community/volunteering, age was not significantly associated with other forms of restriction in this study. Rather, health status indicators such as healthcare interaction, work status/disability, and disease-related emotional/physical problems were observed to restrict social engagement among those living with chronic conditions. These findings may support that many health indicators included in this study were age-related (e.g., fewer older adults are employed, more older adults receive care from others) or simply that common barriers exist among adults living with chronic conditions. Despite age, adults with chronic conditions may encounter competing demands on their time (e.g., participants with more physician visits were more likely to restrict social engagement). Although the number of chronic condition diagnoses often increase with age [34,35], co-morbidity was not significantly associated with social engagement restriction in multivariate analyses (see Table 2). Conversely, the symptoms and ramifications of participants’ conditions were associated with restrictions (e.g., disability, hospitalization, receiving care, emotional/physical problems). This is confirmed in bivariate analyses (see Table 1) where larger proportions of those with more physically symptomatic chronic conditions reported restrictions (e.g., diabetes, arthritis, lung disease). Additionally, a larger proportion of participants with depression also reported social engagement restrictions (confirmed by the significant relationship of the EPPS scores with restrictions in all multivariate analyses) and other studies showing the link between mental health and isolation among older adults [36,37,38]. Future studies that further examine the influence of age and specific disease profiles on social engagement restrictions are encouraged.

Study findings provide insight into interventions for healthy aging. Multiple interventions are necessary to prevent and manage chronic disease while promoting social engagement. Environmental interventions focused on the built environment can be a critical component that can both target social interactions and physical activity [39,40]. Identifying sustainable solutions—namely walkable environments that will help increase or maintain adequate levels of physical activity [12,39,40,41,42]—is critical. Potential solutions also transcend environmental solutions into behavioral interventions.

Behavioral interventions are another area of potential use in targeting chronic disease and related social engagement. For example, a large proportion of participants who reported social engagement restrictions from their disease also indicated requiring assistance to learn how to take care of themselves. This highlights a need for chronic disease self-management education. An ideal solution for improving an individual’s knowledge on chronic disease self-care management is offering and encouraging involvement in the Chronic Disease Self-Care Management Program (CDSMP) [43]. This program is a six-week course aimed at improving individual’s self-efficacy concerning their ability to manage their chronic disease and its effects on their life, improving not only their physical health, but their mental and social health as well [43]. Usually hosted within a community setting, the CDSMP is scientifically supported and can lead to improvements in physical activity and reductions in hospitalizations [44,45].

In addition to learning skills and strategies for self-care, in-person or online participation in chronic disease self-management programs may empower participants to actively engage in life as well as obtain the social support they may need [46]. Engaging in online support groups for individuals with specific chronic conditions is another option that can provide a sense of community and increased social well-being [47]. Further investigation is needed to understand the social networks of adults with chronic conditions [48,49] and their influence on intervention participation and social-related outcomes.

This study had limitations. First, all survey results were based on participants’ self-reported behaviors, which may limit the validity of the findings. Self-reported data included chronic condition diagnoses, which were not confirmed by a clinician or medical report/source. Second, although the NCOA Chronic Care Survey is a nationally representative probability survey of Americans 44 years and older with chronic conditions, results may not be entirely generalizable to middle-aged and older Americans. Third, the internal consistency reliability for the EPPS was somewhat below accepted standards, which may have implications for the strength of relationships observed with this composite scale and other interpretations. Fourth, other than education, there were no indicators of socioeconomic status included in this study, despite income being a known contributor to health and social engagement. It is unknown, for example, if cutting back on helping family and friends related to financial contributions or other forms of support (e.g., study findings show unemployed participants were more likely to restrict social engagement). Lastly, some study analyses examined associations with reporting one or more social engagement restrictions. While this information is valuable, it is also important to consider if participant characteristics are similar or different for those reporting one versus two versus three restrictions. Given the adequacy of sample size and variable distributions, future studies are encouraged to examine such relationships using multinomial, ordinal, and/or linear regression models.

Despite these limitations, this study sheds light on the important finding that middle-aged and older adults with chronic conditions report social engagement restrictions, and that several factors are related to these restricted behaviors. Considering that being socially engaged may result in numerous positive health benefits for healthy aging [9], it is important for aging adults with chronic conditions as well as their family members, friends, health care professionals, neighborhoods and communities on a larger scale to work toward increasing understanding about social engagement restrictions and developing ways to address them.

## 5. Conclusions

Middle-aged and older men and women with one or more chronic conditions may restrict their social engagement with family, friends, and within their community. Several factors are associated with reporting social engagement restrictions, such as higher education, receiving care, having more physician visits and hospitalizations, having a disability, being unemployed, and having higher scores on the Emotional and Physical Problems Scale. Environmental and behavioral interventions can help middle-aged and older adults better manage their chronic conditions and maintain an active social life.

## Figures and Tables

**Table 1:** Personal characteristics by social engagement self-restrictions resulting from health problems.

	Total (n = 793)	Has 1+ Social Engagement Restrictions			
		No (n = 457)	Yes (n = 336)	*X^2^* or *t*	*p*
Social Engagement Restrictions Resulting from Health Problems					
*Results of Health Problems: Cut Down or Skip Social Activities*				-	-
No	69.0%	-	-		
Yes	31.0%	-	-		
*Results of Health Problems: Cut Back on Helping Family and Friends*				-	-
No	73.1%	-	-		
Yes	26.9%	-	-		
*Results of Health Problems: Cut Back on Helping Community/Volunteering*				-	-
No	72.7%	-	-		
Yes	27.3%	-	-		
*Number of Social Engagement Restrictions (possible range 0 to 3)*	0.85 (±1.18)	-	-	-	-
*Age*				0.10	0.749
44–64 years	66.2%	66.6%	65.5%		
65+ years	33.8%	33.4%	34.5%		
*Sex*				1.41	0.234
Male	45.3%	47.0%	42.7%		
Female	54.7%	53.0%	57.3%		
*Race/Ethnicity*				8.16	0.043
Non-Hispanic White	84.0%	86.6%	80.0%		
Non-Hispanic African American	8.9%	7.9%	10.5%		
Hispanic	4.9%	4.2%	6.0%		
Other	2.1%	1.3%	3.5%		
*Education*				13.26	0.010
Less than High School	7.7%	6.9%	8.9%		
High School	34.9%	33.8%	36.6%		
Some College	22.2%	21.1%	23.9%		
College Graduate	22.7%	22.3%	23.2%		
Graduate School	12.5%	15.9%	7.3%		
*Marital Status*				13.86	<0.001
Unmarried	37.8%	32.6%	45.7%		
Married	62.2%	67.4%	54.3%		
*Work Status*				142.55	<0.001
Employed	37.2%	46.6%	22.9%		
Unemployed	12.2%	10.0%	15.6%		
Retired	38.3%	41.5%	33.4%		
Disabled	12.2%	1.9%	28.0%		
*Number of Adults in Household*	1.96 (±0.79)	1.99 (±0.78)	1.93 (±0.81)	0.95	0.342
*Reported Chronic Disease Types (endorsed ‘yes’)*					
Cardiovascular Disease	63.9%	61.8%	67.2%	2.40	0.121
Diabetes	26.3%	22.5%	31.9%	8.64	0.003
Arthritis	49.4%	41.5%	61.3%	29.68	<0.001
Lung Disease	24.8%	20.0%	32.2%	14.93	<0.001
Depression or Anxiety	26.0%	16.7%	40.1%	54.13	<0.001
Cancer	13.0%	11.3%	15.6%	3.15	0.076
*Number of Chronic Condition Types*	2.04 (±1.06)	1.74 (±0.90)	2.49 (±1.12)	–9.89	<0.001
*Number of Physician Visits (past year)*	3.14 (±1.95)	2.54 (±1.70)	4.07 (±1.95)	–11.35	<0.001
*Number of Hospitalizations (past year)*	0.34 (±0.99)	0.15 (±0.66)	0.64 (±1.28)	–6.13	<0.001
*Caregiving Status*				106.47	<0.001
Neither Provide Nor Receive	66.2%	76.0%	51.3%		
Provide Only	21.4%	21.1%	22.0%		
Receive Only	9.5%	1.7%	21.3%		
Both Provide and Receive	2.9%	1.3%	5.4%		
*Get Help & Support Needed to Manage Health Problems*				27.15	<0.001
Never	8.1%	8.1%	8.0%		
Rarely	8.1%	6.7%	10.2%		
Occasionally	23.0%	22.3%	23.9%		
Frequently	30.1%	25.7%	36.9%		
Always	30.8%	37.2%	21.0%		
*Emotional and Physical Problems Scale*	5.03 (±3.64)	3.42 (±2.53)	7.48 (±3.71)	–6.97	<0.001
*I need help learning how to take care of myself*				28.69	<0.001
No	65.3%	72.7%	54.1%		
Yes	34.7%	27.3%	45.9%		

**Table 2:** Factors associated with social engagement restrictions resulting from health problems.

	Has 1+ Social Engagement Restrictions				Cut Back on Helping Family & Friends				Cut Down or Skip Social Activities				Cut Back on Helping in Your Community/Volunteering			
	OR	*p*	95% CI		OR	*p*	95% CI		OR	*p*	95% CI		OR	*p*	95% CI	
			Lower	Upper			Lower	Upper			Lower	Upper			Lower	Upper
Age: 44–64 years	1.00	--	--	--	1.00	--	--	--	1.00	--	--	--	1.00	--	--	--
Age: 65+ years	1.63	0.060	0.98	2.72	0.74	0.281	0.42	1.29	1.28	0.375	0.74	2.20	1.70	0.046	1.01	2.86
Male	1.00	--	--	--	1.00	--	--	--	1.00	--	--	--	1.00	--	--	--
Female	1.10	0.640	0.73	1.66	1.18	0.473	0.76	1.83	1.03	0.883	0.68	1.57	1.85	0.004	1.21	2.82
Non-Hispanic White	1.00	--	--	--	1.00	--	--	--	1.00	--	--	--	1.00	--	--	--
Non-Hispanic African American	1.00	0.992	0.50	2.01	1.36	0.379	0.68	2.73	1.12	0.748	0.56	2.25	1.25	0.509	0.64	2.45
Hispanic	0.80	0.646	0.30	2.10	0.66	0.428	0.24	1.83	0.78	0.619	0.28	2.11	0.97	0.953	0.37	2.53
Other Race	2.19	0.413	0.34	14.30	1.35	0.743	0.22	8.21	1.23	0.802	0.24	6.20	0.85	0.830	0.18	3.93
Less than High School	1.00	--	--	--	1.00	--	--	--	1.00	--	--	--	1.00	--	--	--
High School	3.57	0.005	1.48	8.63	2.00	0.126	0.82	4.88	4.02	0.003	1.59	10.15	3.64	0.003	1.55	8.56
Some College	4.25	0.002	1.68	10.75	2.57	0.048	1.01	6.54	3.85	0.006	1.47	10.13	2.83	0.022	1.16	6.89
College Graduate	4.61	0.001	1.81	11.78	1.94	0.168	0.76	5.01	5.94	<0.001	2.23	15.83	3.76	0.004	1.51	9.33
Graduate School	2.56	0.077	0.90	7.26	1.28	0.673	0.41	4.01	3.72	0.019	1.24	11.15	1.14	0.816	0.38	3.47
Number of Chronic Condition Types	1.18	0.110	0.96	1.46	0.95	0.676	0.76	1.19	1.09	0.443	0.88	1.34	1.18	0.107	0.97	1.45
Number of Physician Visits (past year)	1.26	<0.001	1.12	1.41	1.09	0.145	0.97	1.23	1.34	<0.001	1.19	1.50	1.16	0.010	1.04	1.30
Number of Hospitalizations (past year)	1.25	0.069	0.98	1.60	1.21	0.109	0.96	1.53	1.40	0.005	1.11	1.77	1.26	0.037	1.01	1.56
Unmarried	1.00	--	--	--	1.00	--	--	--	1.00	--	--	--	1.00	--	--	--
Married	0.80	0.363	0.50	1.29	0.75	0.263	0.45	1.24	0.82	0.431	0.50	1.34	1.24	0.386	0.77	2.00
Work Status: Employed	1.00	--	--	--	1.00	--	--	--	1.00	--	--	--	1.00	--	--	--
Work Status: Unemployed	2.42	0.005	1.30	4.49	2.79	0.002	1.46	5.32	1.49	0.215	0.79	2.80	2.17	0.015	1.16	4.06
Work Status: Retired	1.43	0.200	0.83	2.48	2.19	0.013	1.18	4.08	0.99	0.962	0.55	1.77	1.37	0.296	0.76	2.45
Work Status: Disabled	9.58	<0.001	3.67	24.99	10.06	<0.001	4.56	22.20	6.25	<0.001	2.82	13.89	3.23	0.001	1.58	6.61
Number of Adults in Household	0.97	0.845	0.73	1.29	1.16	0.323	0.86	1.56	1.12	0.455	0.84	1.50	0.90	0.488	0.67	1.21
Care Status: Neither Provide Nor Receive	1.00	--	--	--	1.00	--	--	--	1.00	--	--	--	1.00	--	--	--
Care Status: Provide Only	1.39	0.159	0.88	2.20	1.58	0.072	0.96	2.60	1.71	0.025	1.07	2.74	1.38	0.188	0.86	2.21
Care Status: Receive Only	6.10	<0.001	2.40	15.54	5.18	<0.001	2.43	11.06	3.72	0.001	1.75	7.93	3.42	0.001	1.67	6.99
Care Status: Both Provide and Receive	1.30	0.690	0.36	4.66	2.14	0.194	0.68	6.78	2.64	0.123	0.77	9.06	1.31	0.633	0.43	3.95
Get Support: Never	1.00	--	--	--	1.00	--	--	--	1.00	--	--	--	1.00	--	--	--
Get Support: Rarely	1.22	0.681	0.47	3.20	1.29	0.630	0.46	3.61	3.50	0.025	1.17	10.43	1.67	0.296	0.64	4.40
Get Support: Occasionally	1.05	0.910	0.46	2.37	1.34	0.514	0.56	3.21	3.39	0.014	1.28	8.96	1.24	0.614	0.53	2.89
Get Support: Frequently	1.17	0.692	0.53	2.60	1.35	0.490	0.58	3.16	2.77	0.035	1.07	7.16	1.53	0.310	0.67	3.47
Get Support: Always	0.70	0.387	0.31	1.58	1.15	0.755	0.48	2.78	2.07	0.145	0.78	5.52	0.91	0.832	0.39	2.13
Barrier HOW to Manage Condition: No	1.00	--	--	--	1.00	--	--	--	1.00	--	--	--	1.00	--	--	--
Barrier HOW to Manage Condition: Yes	1.36	0.162	0.88	2.08	1.67	0.029	1.05	2.64	1.52	0.058	0.99	2.35	0.87	0.518	0.56	1.34
Emotional and Physical Problems Scale	1.42	<0.001	1.31	1.54	1.36	<0.001	1.26	1.48	1.29	<0.001	1.20	1.39	1.30	<0.001	1.21	1.40
	Nagelkerke R^2^ = 0.556				Nagelkerke R^2^ = 0.522				Nagelkerke R^2^ = 0.519				Nagelkerke R^2^ = 0.449			
